# Editorial: Interfacial Structures and Their Properties

**DOI:** 10.3389/fchem.2021.807066

**Published:** 2021-12-07

**Authors:** Joachim Paier, Peter Broqvist, Xiaohang Lin

**Affiliations:** ^1^ Institut für Chemie, Humboldt-Universität zu Berlin, Berlin, Germany; ^2^ Department of Chemistry – Ångström Laboratory, Uppsala University, Uppsala, Sweden; ^3^ Key Laboratory for Liquid-Solid Structural Evolution and Processing of Materials, Ministry of Education, Shandong University, Jinan, China

**Keywords:** nanoparticles, surfaces, electrochemistry, density functional theory, machine learning

Structure-property relationships of interfaces are immensely relevant not least because they affect our everyday lives crucially (Erdemir, 2005; Rosenberg, 2005). Interface and surface science rapidly develops in many directions currently, both at the fundamental and applied levels. Life sciences (Kumar, 2010), heterogeneous catalysis (Bell, 2003; Freund et al., 2011; Franco et al., 2020; Hanikel et al., 2021), electrochemistry (Gohda et al., 2008; Schnur and Groß, 2009; Zhang et al., 2018; Tang et al., 2020), battery technology (Wang et al., 2018), metallurgy (Seah, 1975; Marchand et al., 2020; Debroy et al., 2021), and organic chemistry (Ma et al., 2010; Li et al., 2014; Delville and Taubert, 2018) are representative examples for active fields, but this list must remain vastly incomplete by obvious reasons. From a fundamental science perspective, however, it is a formidable task to develop clear-cut relationships between actual atomic interfacial structures and related physicochemical properties. The present research topic attempts to collect some of the latest research results associated with the above-mentioned themes. It has been intended to bridge experiment—theory gaps and foster potential collaborations. The editorial is convinced that this represents a viable way to make substantial progress in the domain of interface science.

It is understood that the distribution of reactants affects reactivities. This can be exploited in preparing nano- or micro-structured droplets of solvents. These can be readily monitored by mass-spectrometry. From an experimental point of view, microdroplet reactions appear to have great potential in efficient screening of parameters such as the yield of chemical reactions (Yang et al.). Examined these possibilities for the synthesis of quinoxaline derivatives recently. The synthesis via a microdroplet technique offers substantially shorter reaction times, simpler operation, significantly enhanced yields, and this technique is also environmentally friendlier, because one can go for the reaction without the catalyst. These points represent clear advantages over traditional bulk-phase synthetic strategies.

Central to applications in metallurgy is the atomic-level understanding of melts. There exists consensus in the community that (high temperature) metallic melts contain atomic clusters, but how these clusters evolve under given conditions like temperature and pressure is still unclear (Lou et al., 2013). Along these lines (Song et al.), carefully examined the atomic structure of Al and Cu cluster by virtue of thermodynamic Wulff constructions (based on density functional theory results) and the average cluster size (by pair-distribution functions of observed high-temperature X-ray diffraction (HTXRD) results). Theoretical XRD pattern matched the experimental ones quantitatively in terms of peak positions and widths including relative intensities. This represents a showcase in terms of developing fundamental understanding of a complex network of processes such as the ones occurring in metallic melts.

The electrochemical interface, most often the interface between aqueous solutions and metal surfaces, is at the heart of electrocatalytic applications (Gossenberger et al.). Applied a grand-canonical approach (Groß, 2021) to study the stabilities of sulfate and bisulfate ions in water adsorbed on the (111) surfaces of Pt and Au. The study shows that quantum chemical calculations based on the computational hydrogen electrode, which includes the electrochemical environment in an appropriate way, can reliably predict the stable adsorbate phases at electrochemical electrode/electrolyte interfaces as a function of electrochemical control parameters.

Artificial intelligence or machine learning is expected to push frontiers in the modelling of complex structures and their corresponding properties (Li et al.). studied the transferability and performances of machine-learned force fields of complex metal oxides like magnetite (Fe_3_O_4_) and water adsorbed on the (1 × 1)-(111) surface containing up to four water molecules per unit cell. Approximations involved in the construction of the machine-learned force fields, i.e., missing long-range and incomplete many-body short-range interactions, as well as the electronic structure method underlying the training runs, will critically affect accuracy. They concluded that more work must be spent to relieve these limitations of machine-learned force fields when applied to complex, hydrogen-bond interactions on reducible oxides.

The editors of the present research topic are convinced that it represents an outline of important contributions from the various fields indicating the great interest in related developments and collaborations between experimentalists and theorists. Furthermore, they believe that many more developments will appear in upcoming years triggering important technical applications, including, e.g., catalysis and energy-related applications.
